# Quantified Mechanical Properties of the Deltoid Muscle Using the Shear Wave Elastography: Potential Implications for Reverse Shoulder Arthroplasty

**DOI:** 10.1371/journal.pone.0155102

**Published:** 2016-05-06

**Authors:** Taku Hatta, Hugo Giambini, Koji Sukegawa, Yoshiaki Yamanaka, John W. Sperling, Scott P. Steinmann, Eiji Itoi, Kai-Nan An

**Affiliations:** 1 Biomechanics Laboratory, Division of Orthopedic Research, Mayo Clinic, Rochester, Minnesota, United States of America; 2 Department of Orthopedic Surgery, Mayo Clinic, Rochester, Minnesota, United States of America; 3 Department of Orthopaedic Surgery, Tohoku University School of Medicine, Sendai, Japan; Universite de Nantes, FRANCE

## Abstract

The deltoid muscle plays a critical role in the biomechanics of shoulders undergoing reverse shoulder arthroplasty (RSA). However, both pre- and postoperative assessment of the deltoid muscle quality still remains challenging. The purposes of this study were to establish a novel methodology of shear wave elastography (SWE) to quantify the mechanical properties of the deltoid muscle, and to investigate the reliability of this technique using cadaveric shoulders for the purpose of RSA. Eight fresh-frozen cadaveric shoulders were obtained. The deltoid muscles were divided into 5 segments (A1, A2, M, P1 and P2) according to the muscle fiber orientation and SWE values were measured for each segment. Intra- and inter-observer reliability was evaluated using intraclass correlation coefficient (ICC). To measure the response of muscle tension during RSA, the humeral shaft was osteotomized and subsequently elongated by an external fixator (intact to 15 mm elongation). SWE of the deltoid muscle was measured under each stretch condition. Intra- and inter-observer reliability of SWE measurements for all regions showed 0.761–0.963 and 0.718–0.947 for ICC(2,1). Especially, SWE measurements for segments A2 and M presented satisfactory repeatability. Elongated deltoid muscles by the external fixator showed a progressive increase in passive stiffness for all muscular segments. Especially, SWE outcomes of segments A2 and M reliably showed an exponential growth upon stretching (R^2^ = 0.558 and 0.593). Segmental measurements using SWE could be reliably and feasibly used to quantitatively assess the mechanical properties of the deltoid muscle, especially in the anterior and middle portions. This novel technique based on the anatomical features may provide helpful information of the deltoid muscle properties during treatment of RSA.

## Introduction

Reverse shoulder arthroplasty (RSA) is a common surgical option in patients with severe shoulder pathologies, including cuff tear arthropathy, end-staged osteoarthritis, comminuted proximal humeral fractures, and failed shoulder arthroplasties [1, 2]. With its characteristic reconstruction reversing the anatomical geometries of the glenohumeral joint, RSA allows for a decrease in pain and improvement of shoulder range of motion (ROM), especially elevation [3, 4]. Despite its promising results, there exists a substantial variability regarding implant combination, leading to varied clinical outcomes after RSA [5, 6]. Pre- and postoperative conditions of the deltoid muscle have been identified as key factors affecting surgical outcomes, as this muscle generates glenohumeral elevation solely after RSA [5, 7–10]. In particular, excessive tension in the deltoid muscle after RSA may be associated with subsequent pain, restricted motion, or complications such as acromion fracture and rupture or chronic failure of the deltoid muscle [1, 2, 5, 11, 12]. On the contrary, if the deltoid muscle presents insufficient tension, unsatisfactory outcomes include decreased strength for shoulder motion or postoperative instability [5, 9]. In some cases undergoing RSA, however, difficulties exist in determining the optimal condition (*e*.*g*. tension, stiffness) for the deltoid muscle during the pre- or intraoperative assessment. To date, surgeons need to select the appropriate size and/or combination of RSA implants based on experience in order to assess the tension and stability in replaced joints.

Shear wave elastography (SWE), a novel ultrasound technique, has been a recent focus for quantification of the mechanical properties of various soft tissues. Several studies have used this technique to assess passive stiffness of skeletal muscles in association with various muscular conditions or pathologies [13–17]. To our knowledge, however, there have been no studies using this technique to assess the deltoid muscle. We hypothesized that this technique could be a helpful tool to 1) quantify the mechanical properties of the deltoid muscle during preoperative planning before performing RSA, 2) determine the optimal implant size or combination to achieve close to normal muscle properties, and 3) implement during post-operative rehabilitation to monitor muscle properties.

Skeletal muscle applications using SWE require the ultrasound probe to be placed parallel to the muscle fiber orientation [14, 16, 18]. We have previously reported the anatomical features of the supraspinatus muscle for SWE measurements by dividing the muscle into four muscular segments according to the fiber orientation [15]. For the assessment of large muscles such as the deltoid muscle, we therefore needed to establish a feasible imaging methodology based on the anatomical features due to variable fiber orientation within the muscle. Classically, the deltoid muscle has been previously divided into three portions based on muscular activity and/or its function; anterior (clavicularis), middle (acromialis) and posterior (spinalis) [5, 7–10, 19–21]. On the other hand, Sakoma et al. [22] differentiated seven segments based on the orientation of the intramuscular tendon. The study also showed, using positron emission tomography, that the lateral three segments originating from the lateral side of the acromion (trisected into anterior, middle, and posterior) presented various activity patterns and mainly acted on shoulder abduction. We attempted to assess 5 muscular regions independently, corresponding to the clavicularis, three parts of the acromialis, and the spinalis.

The purposes of this study were 1) to determine the feasible placement of the ultrasound probe for SWE imaging according to muscle fiber orientation on the deltoid muscle regions, and 2) to investigate the reliability and validity of this technique using cadaveric shoulders.

## Materials and Methods

### Specimen Preparation

**Fig 1:**
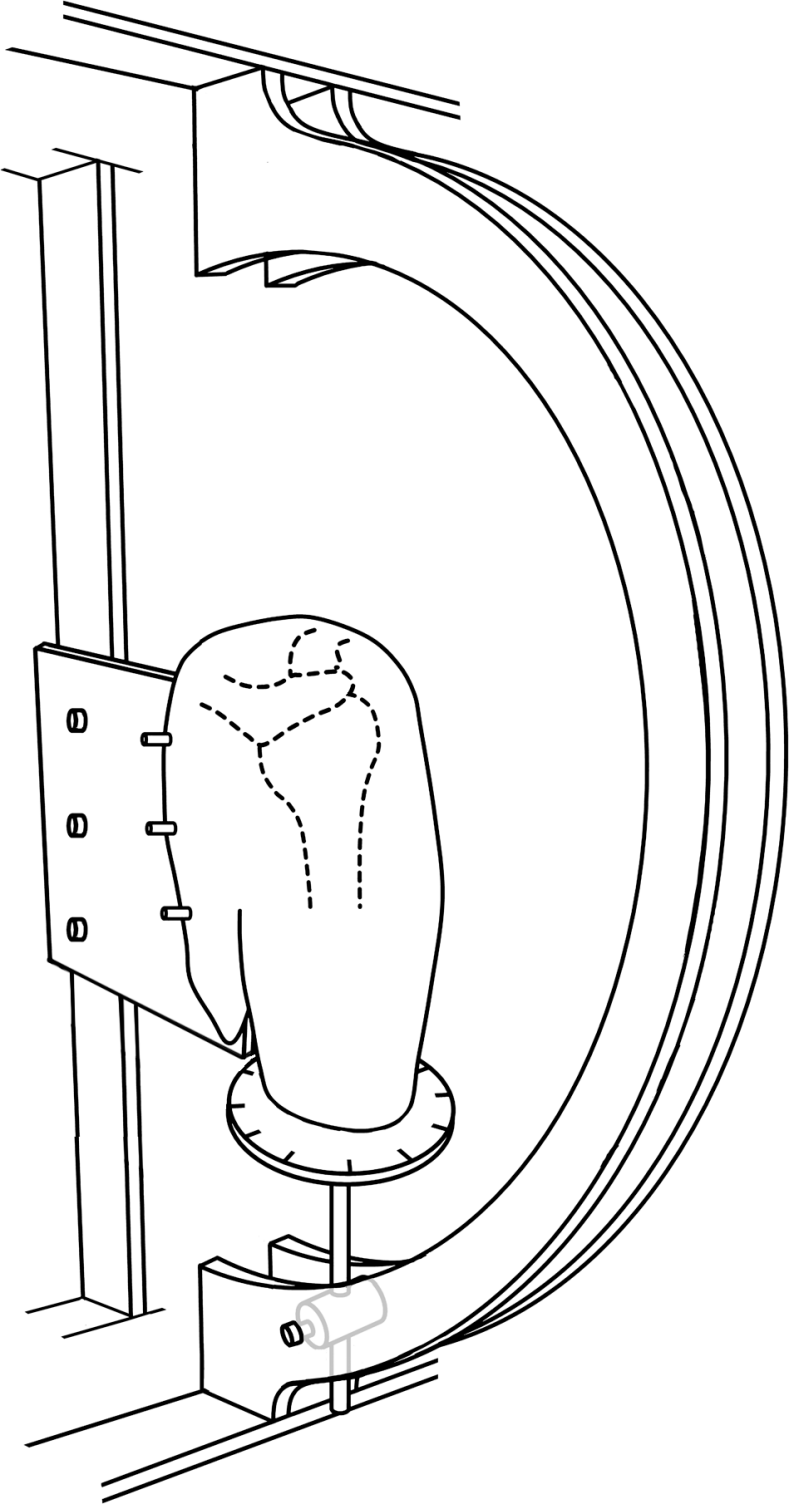
Schematic of the shoulder experimental custom-made device.

Eight (8) fresh-frozen shoulders from 8 human cadavers were obtained from the Mayo Clinic Anatomy Department after internal approval from the Mayo Biospecimens Subcommittee. Written informed consent was obtained from the donor before the start of this research. The mean age at death was 83 years (range, 72–90 years). Before the experiment, the specimens (preserved at −20°C in a freezer) were thawed overnight at room temperature (24°C). The scapula was disarticulated from the thorax, and the humerus was cut at the level of the midshaft, maintaining the distal attachments of the deltoid muscle. The scapula and a fiberglass rod inserted into the humeral medullary canal were both secured in a custom-designed experimental device. According to the International Society of Biomechanics (ISB) recommendation and relevant studies, the scapula was secured at 0° of upward/downward rotation considered as a neutral position [23, 24]. The custom-designed experimental device is designed to provide 6 degrees-of-freedom motion of the glenohumeral joint in consistent motion paths [25] (Fig 1). In this study, cadavers were placed with the shoulder positions of 0° abduction and 0° rotation.

### Shear Wave Elastography

**Fig 2:**
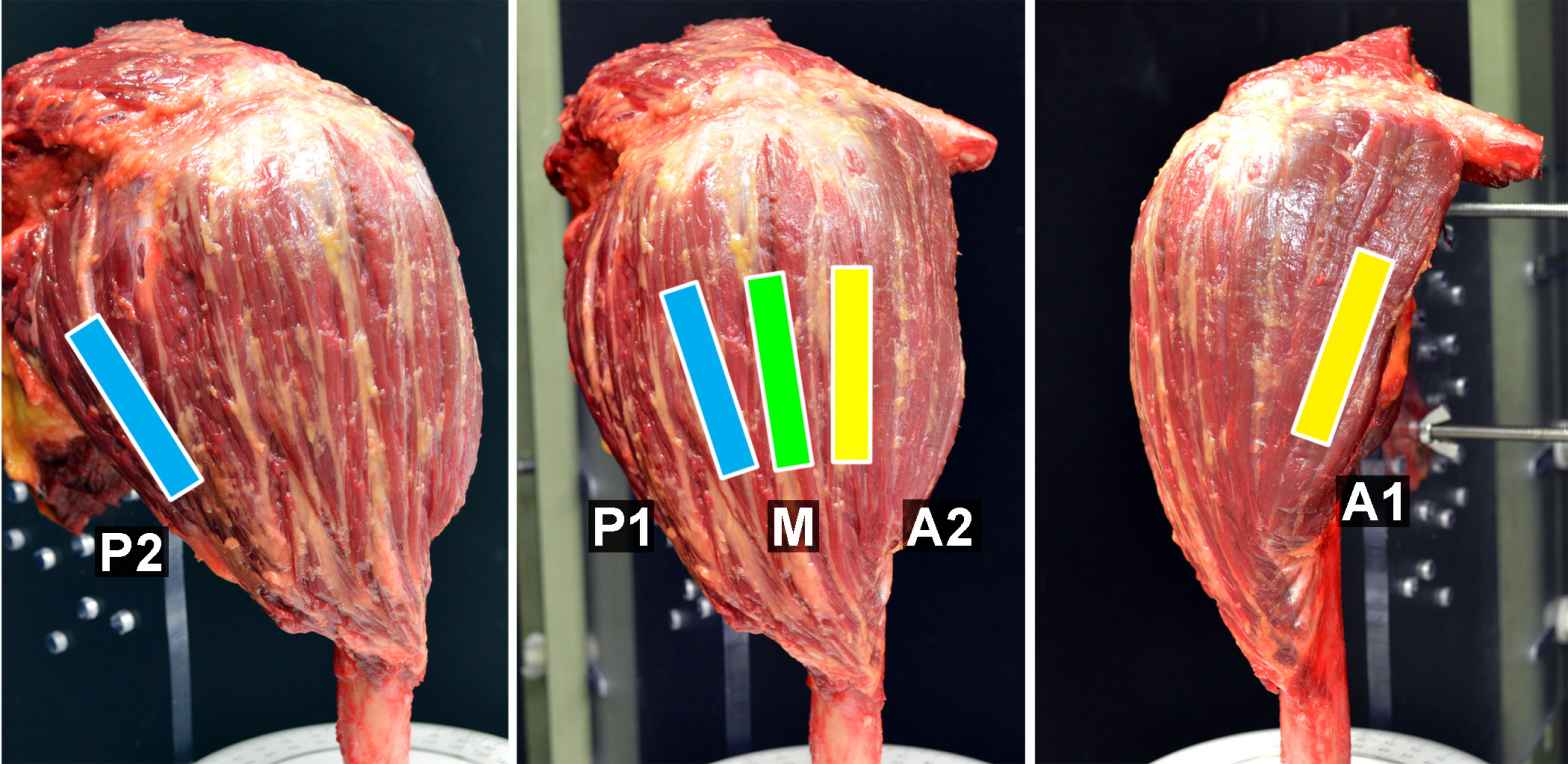
Deltoid muscle anatomy for positioning of ultrasound probe during SWE measurements. SWE was examined percutaneously and the values were obtained from 5 segments; anterior (A1, A2), middle (M), and posterior (P1, P2).

An ultrasound system (Aixplorer; Supersonic Imagine, Ltd., Aix-en-provence, France) and a linear array probe (SL10-2; Supersonic Imagine, Ltd.) (center frequency 6 MHz, pitch 0.2 mm, 192 elements, bandwidth 80%, elevation focus at 30 mm) were used to perform the ultrasound examinations. SWE was examined percutaneously, and images for the SWE measurements were obtained from 5 muscular segments divided according to the muscle fiber orientation; anterior (A1, A2), middle (M), and posterior segments (P1, P2). SWE values for each segment were assessed independently on the plane parallel to the muscle fibers (Fig 2). Initially, proximal and distal attachments of the deltoid muscle were identified sonographically, and the midpoint level of the muscle belly was determined for the SWE measurements. To assess the A1 (clavicularis) and P2 (spinalis) regions, the probe was positioned 10 mm inside from the anterior or posterior margins of the muscle. Muscle fibers from the A2, M and P1 regions were identified as those originating from the anterolateral corner, midpoint, and posterolateral corner (acromial angle) of the acromion, respectively. In order to avoid any artifact in SWE measurements, the ultrasound probe was placed in the muscular region avoiding the intramuscular tendon. Using a built-in-software, SWE values corresponding to the elastic modulus (kPa) were obtained for each segment. In order to minimize the technical variation arising from probe positioning or probe pressure, SWE values were measured repeatedly 9 times as previously described for elastographic assessments [15, 26]. Briefly, the ultrasound transducer was positioned on the muscle of interest, data was acquired, and the transducer was then lifted from the muscle before an additional measurement was performed. This process was repeated 9 times. The mean SWE values were then calculated for all segments from these images to obtain the elastic modulus of the muscle segments.

Three investigators (TH, KS, and YY) measured SWE values independently. One investigator (TH) repeated the measurements twice within one-hour interval to assess intra-observer reproducibility. Thus, intra- and inter-observer reliability were evaluated on the current SWE technique for measuring deltoid muscle elasticity.

**Fig 3:**
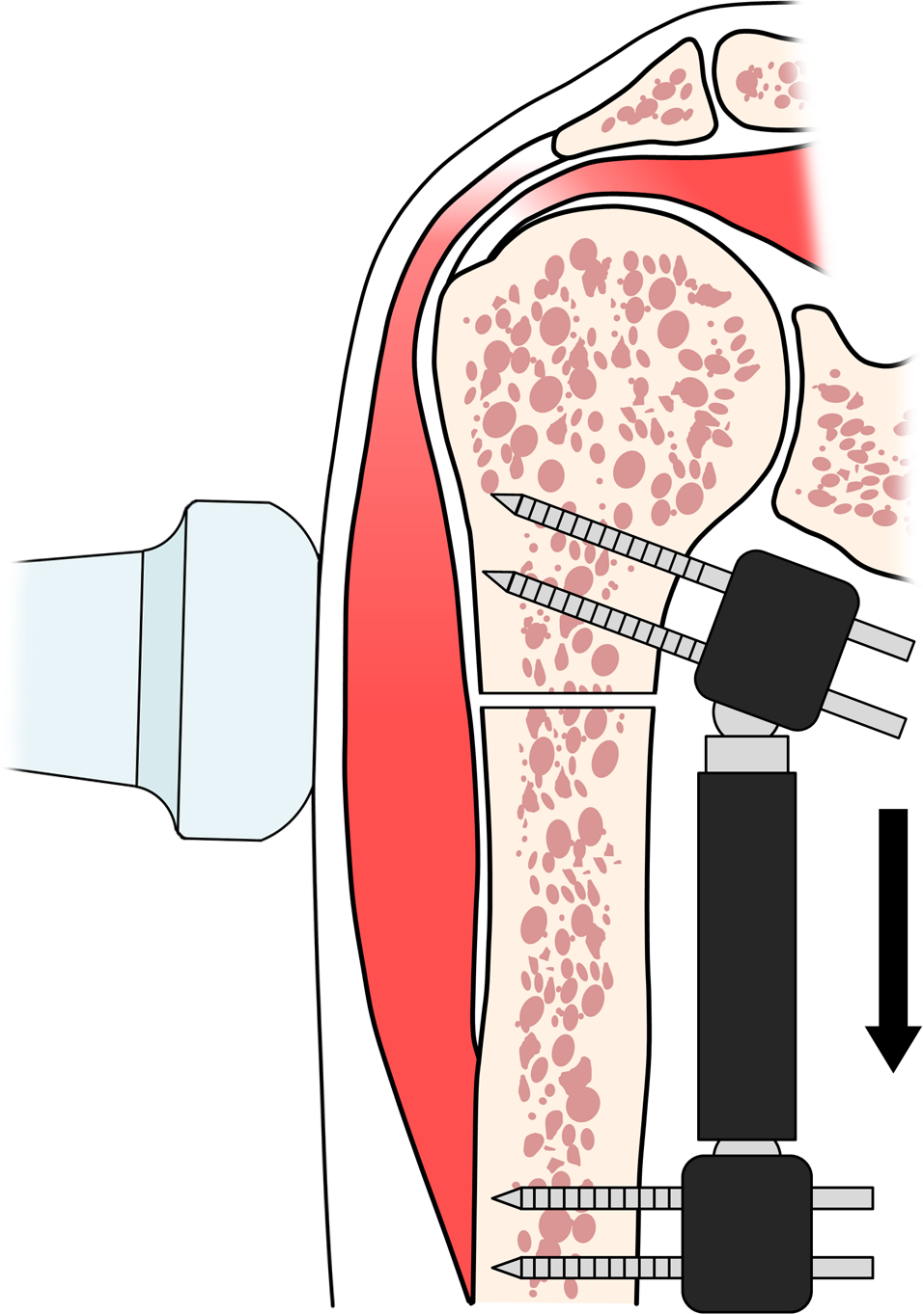
Experimental elongation of the deltoid muscle. Tensile strain in the muscle was generated with the external fixator (arrow). SWE probe was placed at the midpoint level of the deltoid muscle. SWE was examined with intact length (0 mm), and under elongated conditions (+5, +10, and +15 mm).

**Fig 4:**
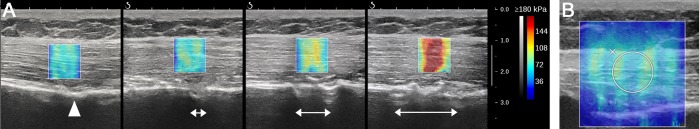
A) SWE images of elongated deltoid muscle. The colored regions represent the SWE modulus map with the scale to the right of the figure. Arrow head represents the osteotomized region without elongation (0 mm). Arrows represent the extent of elongation (+5, +10, and +15 mm). B) A circular region of interest (ROI) was used to obtain SWE values that included the entire thickness of the muscle.

To assess the feasibility of this technique, we modified the mechanical environment of the deltoid muscle by elongating it, potentially altering its properties due to excessive tensile strain. Elongation of the muscle along the humeral axis was achieved with an external fixator (Radiolucent Wrist Fixator, Orthofix Orthopedics International, Ltd., Bussolengo, Verona, Italy, Fig 3). We compared SWE measurements in all segments of the deltoid muscle after humerus osteotomy (0 mm) with those under elongated conditions (+5, +10, and +15 mm, Fig 4). Previous biomechanical studies have demonstrated that a piecewise exponential model could be applied to the passive tension-length relationship of the skeletal muscles [27–29]. In addition, SWE values obtained during muscular elongation or relative joint angles have been demonstrated to fit this behavior [14, 17]. Therefore, we also assessed if the obtained SWE measurements of each segment during elongation of the deltoid muscle represented an exponential behavior.

### Statistical Analyses

Intra- and inter-observer reliability was examined using intraclass correlation coefficient (ICC(2,1). Continuous variables of SWE data were tested for normality and equal variance before performing statistical analyses. Because the data did not present a normal distribution, non-parametric tests were performed. Friedman with Dunn’s post hoc tests were used to evaluate differences in SWE values of the deltoid muscle under intact and elongated conditions (0, +5, +10, and +15 mm). Statistical analyses were performed using the software SPSS (version 18.0, SPSS, Chicago, IL) and GraphPad Prism (version 6.0, GraphPad Prism, San Diego, CA). The significance level was set to p < 0.05.

## Results

Intra- and inter-observer reliability for all segments of the muscle were 0.761–0.963 and 0.718–0.947 for ICC(2,1) (Table 1). In particular, good to excellent reliability was consistently observed in A2 and M segments of the muscle.

**Table 1:** Reliability of shear wave elastography (SWE).

	Intra-observer	Inter-observer
**A1**	0.761 [0.155–0.948]	0.848 [0.601–0.964]
**A2**	0.963 [0.829–0.993]	0.947 [0.832–0.988]
**M**	0.898 [0.607–0.978]	0.884 [0.674–0.973]
**P1**	0.799 [0.252–0.957]	0.773 [0.446–0.943]
**P2**	0.789 [0.300–0.953]	0.718 [0.332–0.929]

Values represent intraclass correlation coefficient (ICC) [95% confidence interval]. ICC(2,1) was used for intra- and inter-observer reliability.

**Fig 5:**
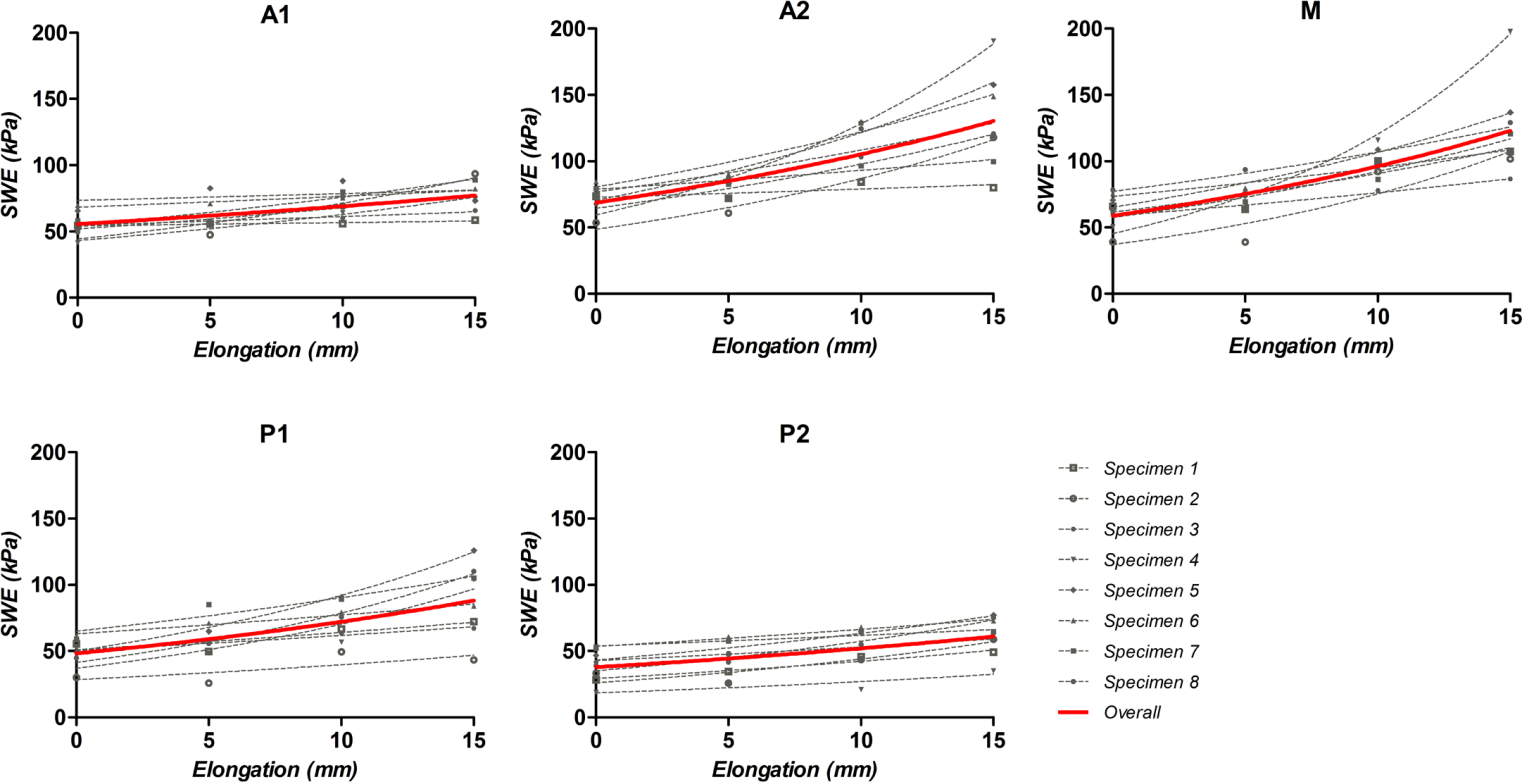
Distribution of SWE values obtained from deltoid muscles with and without elongation. Overall data for each muscular segments were fit using exponential growth curves with R^2^ = 0.390 for A1, 0.558 for A2, 0.593 for M, 0.421 for P1, and 0.306 for P2.

Elongated deltoid muscles with the external fixator showed a progressive increase in passive stiffness for all muscular segments (Table 2). In A2, M and P1, SWE values increased two-fold at 15 mm elongation compared to those at the original length (0 mm). Among the five segments of the deltoid muscle, SWE data of segments A2 and M from 0 to 15 mm elongation were reliably fit by an exponential function with R^2^ = 0.558 and 0.593, respectively (Fig 5).

**Table 2:** SWE values (mean [kPa] ± SD) associated with elongated conditions.

	0 mm	+ 5 mm	+ 10 mm	+ 15 mm
**A1**	55.9 ± 8.9	60.3 ± 11.1	71.2 ± 10.8 ^a^	76.2 ± 11.6 ^b, d^
**A2**	72.4 ± 9.1	77.6 ± 9.8	109.9 ± 20.0 ^a^	129.3 ± 34.9 ^c, d^
**M**	63.0 ± 13.1	69.7 ± 15.7	97.4 ± 12.1 ^a^	123.5 ± 33.9 ^c, e^
**P1**	50.2 ± 9.9	57.2 ± 17.5	71.4 ± 14.9 ^a^	89.0 ± 27.1 ^c, e^
**P2**	39.1 ± 11.9	42.8 ± 13.3	52.2 ± 15.4 ^a^	61.3 ± 14.4 ^c, d^

Friedman test was used to compare SWE values of the elongated deltoid muscle. Significant differences were observed for + 10 mm (**a:** p < 0.05) and + 15 mm (**b:** p < 0.01, **c:** p < 0.001) compared to 0 mm; and for + 15 mm (**d:** p < 0.05, **e:** p < 0.01) compared to + 5 mm.

## Discussion

To our knowledge, this is the first elastographic study focusing on the mechanical properties of the deltoid muscle with implications in reverse shoulder arthroplasty. The current study established a SWE technique for the deltoid muscle based on anatomical and functional characteristics. We have assessed 5 muscular regions independently, A1, A2, M, P1 and P2, corresponding to the clavicularis, three parts of the acromialis, and the spinalis, respectively. This technique provided intra- and inter-observer reliability over 0.72 of ICCs for all muscular segments; especially in segments A2 and M, ICCs showed satisfactory reliability with 0.88–0.96. In contrast, SWE measurements for segments A1, P1 and P2 showed a wider range. These inconsistencies in outcomes among the segments could be explained by the amount of subcutaneous tissue, which could be thicker in the latter 3 segments. A recent study [15] has demonstrated overlying soft tissues above a deeper muscle (e.g. skin, subcutaneous fat) to not directly affect SWE values of the imaged muscle. However, thicker subcutaneous tissues might potentially lead to technical variations relating a consistent placement of the ultrasound probe in the tissue.

The current study showed increased SWE values when more tension was applied to the deltoid muscles by the external fixator. These findings suggest that alteration of mechanical properties on the muscle could clearly be reflected on SWE outcomes. Similar SWE studies for skeletal muscles in the upper [14] or lower extremities [17] in the presence or absence of tensile strains have been carried out to validate the imaging measurements. Based on previous studies and current results, we believe the implemented SWE technique could be reliably used for the deltoid muscle under various conditions.

The importance of deltoid muscles in achieving satisfactory outcomes after RSA has been investigated from clinical observation [30], biomechanical studies using cadaveric shoulders [5, 10, 31], finite element analysis [32], and three-dimensional motion analysis using virtual shoulder models. [7–9, 33]. These biomechanical studies have mostly focused on the altered moment arm of the deltoid muscles, or its required force for shoulder motions. Particularly, the middle portion (acromialis) of the deltoid muscle has been known to alter its mechanical environment during shoulder elevation dramatically after RSA [9]. The segmental measurements in this study demonstrated stiffness variations within the deltoid muscle under a non-stretched condition. Such a variation was also found in other muscles [34, 35], suggesting a possible difference in fiber tension along the muscle thickness and different muscle regions corresponding to varying physiological functions. More detailed analyses of RSA including the type of glenosphere (diameter and lateral offset) [8–10], humeral component (offset and rotation) [5], and their positioning (tilt and rotation) [7, 33] have demonstrated to affect the mechanical properties of the deltoid muscle. In addition to these biomechanical evidences, quantitative SWE assessment would help clarify the changes in mechanical properties of the deltoid muscle after RSA.

In patients with shoulder pathologies, deltoid muscle conditions could be highly individualized. At the advanced stage of cuff tear arthropathy or malunited proximal humeral fractures, the deltoid muscle might be shorten due to upper-migrated/collapsed humeral head, or shortening/angular deformity of the humeral neck. Chronic progress may also increase the difficulty for assessing the appropriate condition of the deltoid muscle preoperatively or intraoperatively. Therefore, we believe that this noninvasive SWE technique could be a useful tool for quantitative assessment of the deltoid muscle properties in addition to the traditional radiologic modalities used for the muscle quality evaluation such as MRI. In addition, intraoperative SWE assessment measured under loosen-stretched conditions may provide more detailed information of the slack angle of the muscle and its mechanical responses. These findings, obtained from individual patients, might be helpful to determine the optimal condition of their deltoid muscle after placement of prosthesis.

There are several limitations in this study. First, SWE data for the deltoid muscle were obtained from cadaveric shoulders. It should be noted that fresh-frozen shoulders might present different muscular responses to elongation when compared to live subjects. In addition, the sample size was small to determine standard values for deltoid properties. Nevertheless, using this methodology, future investigations including more samples and live subjects could define SWE patterns with and without shoulder pathologies. Second, a recent study by Koo and Hug revealed muscle shear modulus could be affected by mechanical, material, and architectural properties of the muscles [36]. Therefore, further *in vivo* studies would be useful to also determine if the SWE values, shear moduli, of the deltoid muscle correlate with deltoid muscle function. Third, we assessed the passive stiffness of the muscle corresponding to shoulder abduction. Further studies with additional shoulder motions should be carried out to determine compartmental differences in SWE outcomes and the relation to shoulder motion. In addition, a comparison of SWE outcomes among muscles of the shoulder joint will provide valuable information to further understand shoulder muscle biomechanics. Fourth, our established methodology for the deltoid segmental measurements advocated the placement of the ultrasound probe independently of any anatomical variation between subjects. Although the methodology is simple and reproducible, future studies should investigate variations in muscle volume to address the optimal region for placement of the probe and any effect and correlation between transducer pressure on the muscle and SWE outcomes.

This study demonstrates a first step toward the assessment of the mechanical properties of the deltoid muscle using SWE. This novel technique based on the anatomical features of the deltoid muscle may provide a useful assessment tool for quantitative assessment of the mechanical condition of the muscle in the clinical practice, especially for the treatment of RSA.

## Conclusions

Shear wave elastography is a reliable and feasible tool for the quantitative assessment of the mechanical properties of the deltoid muscle, especially for anterior and middle segments. Segmental measurements according to the anatomical features might provide characteristic patterns of deltoid muscle properties.
